# Metabolic, Endocrine, and Cardiovascular Risks in Children with Overnutrition and Undernutrition

**DOI:** 10.3390/children9070926

**Published:** 2022-06-21

**Authors:** Valeria Calcaterra, Gianvincenzo Zuccotti

**Affiliations:** 1 Department of Internal Medicine, University of Pavia, 27100 Pavia, Italy; 2 Department of Pediatrics, “Vittore Buzzi” Children’s Hospital, 20157 Milano, Italy; gianvincenzo.zuccotti@unimi.it; 3 Department of Biomedical and Clinical Science, University of Milano, 20157 Milano, Italy

Malnutrition is a severe public health issue for both children and adults [1]. It refers to deficiencies or excesses in nutrient intake, an essential nutrient imbalance, or impaired nutrient utilization [2].

The double malnutrition burden consists of both undernutrition (wasting, stunting, underweight, and mineral- and vitamin-related malnutrition) and overnutrition (being overweight, obesity, and diet-related noncommunicable diseases) [2]. Regarding children under 5 years of age, the World Health Organization (WHO) reported that 155 million are stunted, 52 million are wasted, 17 million are severely wasted, and 41 million present overweight and/or obesity [1].

Child growth is internationally recognized as a crucial indicator of nutritional status and health in various populations. There is a growing body of evidence suggesting that nutritional status can both positively and negatively modulate the organ systems involved in body homeostasis and development, thereby influencing the health risk [3]. Thus, malnourished children represent a “fragile” population and have an increased vulnerability as individuals to adverse health outcomes, including metabolic derangement, compared to others of the same age [4].

A synergistic effect of nutrition and metabolism on human health has been described. In fact, nutrition represents the process of nutrient acquisition from the environment, and metabolism is the process of transforming nutrients into substrates [5]. Both undernutrition and overnutrition can alter homeostatic interactions between nutrition and metabolism, leading to metabolic dysregulation.

In the context of metabolic homeostasis, body composition data may provide further information on metabolic capacity and load; for example, muscle mass in relation to fat mass, visceral obesity, ectopic fat accumulation (e.g., in the liver, pancreas, and kidney), and muscle mass loss are much more strongly predictive of metabolic and endocrine disorders.

The adipose tissue (AT) is considered an endocrine organ and can produce and release biologically active compounds involved in obesity-associated chronic inflammation and pathological metabolic processes [6]. In fact, in the presence of a positive energy balance, the AT undergoes morphological and metabolic changes leading to a massive release of pro-inflammatory cytokine-generating signals that recall the immune cells in place and that consequently result in an increase in the inflammatory infiltrates in the AT, hence the onset of chronic low-grade inflammation [7]. The initiating events of inflammation start early in childhood and modulate interconnected biochemical molecular pathways, such as stress adaptation, epigenetics, inflammation, macromolecular damage, metabolism, proteostasis, and stem cell and tissue regeneration, leading to comorbidities including diabetes, cardiovascular diseases, obesity, hypertension, hyperlipidemia, reduced reproductive capacity, and various cancers and related morbidity and mortality risks [7].

However, the skeletal muscle system is the largest organ in the human body, and not only is it important for mobility and strength, but it also acts as a regulator of metabolic processes and stores macronutrients [8]. In fact, the skeletal muscle secretes a variety of biologically and metabolically active polypeptide factors (myokines), providing coordination between whole-body physiology and energy balance. In patients with chronic diseases, factors such as immobility, malnourishment, hormonal alterations, poor blood flow to the muscle, endothelial dysfunction, and therapy can compromise muscular homeostasis [9]. Consequently, proteolytic processes and systemic inflammation can cause relevant skeletal muscle depletion, leading to dysmetabolism.

Long-lasting adverse malnutrition effects in early life can also be attributed to interconnected biological pathways involving an imbalance in the gut microbiome. As reported by Iddrisu [9], the gut microbiota of children is influenced by diet, which, in turn, can impact a child’s nutritional status. The difference in the gut microbiome between lean and obese subjects has been well described. There might be particular gastrointestinal microbiomes that impart resilience to or present a risk of both undernutrition and overnutrition; the manipulation of the gut microbiota may alleviate the complications related to malnutrition [10].

Thus, it is important to consider that different conditions of pediatric malnutrition, including childhood obesity, prenatal malnutrition, and undernutrition and overnutrition in children with disabilities and/or other chronic diseases, such as inflammatory bowel diseases, chronic respiratory disorders, and cancers, are characterized by an altered body composition and a high metabolic load on a depleted capacity for homoeostasis [2], and they may be strongly associated with a functional decline across multiple inter-related systems, as well as with resistance to stressors, which can cause vulnerability [4,11,12] to the risk of cardiovascular disease, diabetes, metabolic syndrome, endocrine disorders, immune dysfunction, and chronic inflammation. 

Undernutrition and overnutrition have been usually approached as separate public health issues; however, the new emergent reality is that the two forms of malnutrition are interconnected. Therefore, double-duty actions that simultaneously address more than one dimension must be supported for effective policy solutions [2]. 

The early detection of comorbid conditions related to undernutrition and overnutrition is useful for developing personalized interventions and preserving health into adulthood. An appropriate nutritional status that can maintain metabolic homeostasis and regular physical activity to protect skeletal muscle mass are key elements for health and well-being, and for reducing endocrine, metabolic, and cardiovascular disease risks, particularly at the pediatric age. 
